# CDH3 in human tumors: tumor biology and potential therapeutic target

**DOI:** 10.3389/fphar.2026.1874056

**Published:** 2026-06-22

**Authors:** Xu-Sheng Liu, Zhi-Jun Pei

**Affiliations:** Department of Nuclear Medicine, The Second Affiliated Hospital of Soochow University, Suzhou, Jiangsu, China

**Keywords:** biomarker, cancer biology, CDH3, cell adhesion, liquid biopsy, P-cadherin, targeted therapy, translational oncology

## Abstract

Cadherin-3 (CDH3), which encodes P-cadherin, is a classical cadherin involved in epithelial architecture, basal-cell identity, and tissue repair. Once viewed primarily as a structural adhesion molecule, CDH3 is now recognized as a context-dependent regulator of tumor progression and a candidate therapeutic target across several human malignancies. Available evidence indicates that CDH3 is frequently overexpressed in breast, lung, gastric, colorectal, pancreatic, biliary, ovarian, and glioblastoma settings, where it is commonly linked to high grade, invasion, metastasis, therapy resistance, and unfavorable outcomes. A unidirectional interpretation is nevertheless insufficient. In melanoma, bladder carcinoma, hepatocellular carcinoma, renal cancer, and selected lineage-specific settings, CDH3 loss, cytoplasmic redistribution, or restoration can instead associate with altered adhesion control, reduced invasion, or bidirectional phenotypes. Mechanistically, CDH3 operates at the intersection of cell-cell adhesion and signaling output. Its dysregulation is shaped by promoter methylation, chromatin remodeling, transcription-factor programs, noncoding RNAs, oxidative stress, and protein-stability pathways. Functionally, CDH3 can amplify epidermal growth factor receptor and insulin-like growth factor 1 receptor signaling, cooperate with Wnt and Rho GTPase circuits, organize collective migration and extracellular-matrix remodeling, support stem-like and metabolic states, and integrate hypoxia- or exosome-driven communication. Translationally, CDH3 has progressed from a tumor-associated antigen to a platform for monoclonal antibodies, antibody-drug conjugates, bispecific T-cell engagers, radioimmunotherapy, and apoptosis-inducing bispecific constructs. Clinical implementation remains constrained by expression heterogeneity, normal-tissue expression, and the lack of robust companion diagnostics. Clarifying its lineage dependence, membrane-functional state, and biomarker-guided therapeutic window will be essential for converting promising biological knowledge into clinically useful diagnostics and treatments.

## Introduction

Cadherins occupy a central position in cancer biology because they link tissue architecture to cell-state plasticity. Early models emphasized loss of epithelial cadherins, especially E-cadherin, as a prerequisite for invasion and metastasis. Cadherin behavior in human cancer is more complex than a simple loss-of-adhesion model. Different cadherins can be co-expressed, switched, redistributed, or functionally rewired according to lineage identity, microenvironmental cues, and oncogenic signaling programs. CDH3, which encodes P-cadherin, illustrates this complexity particularly well. Initially regarded mainly as a marker of basal epithelial compartments and placental tissue, it is now linked to cancer progression, collective migration, metabolic adaptation, biomarker development, and cell-surface targetability across multiple tumor types (Paredes et al., 2005; Albergaria et al., 2011; Siret et al., 2018).

Several observations explain the growing translational interest in CDH3. Clinicopathological studies repeatedly associate CDH3 overexpression with high-grade disease, aggressive phenotypes, and poor survival in breast cancer, non-small cell lung cancer, colorectal cancer, pancreatic cancer, cholangiocarcinoma, ovarian cancer, and glioblastoma (Paredes et al., 2005; Turashvili et al., 2011; Sakamoto et al., 2015; Imai et al., 2018; Martins et al., 2022; Canário et al., 2025). Mechanistic work indicates that P-cadherin is not simply a passive adhesion marker. CDH3 can reshape receptor tyrosine kinase signaling, coordinate Rho GTPase-dependent mechanics, drive leader-cell behavior during collective invasion, and support stem-like, glycolytic, or anoikis-resistant states (Lysne et al., 2014; Sousa et al., 2014; Plutoni et al., 2016b; Le Borgne-Rochet et al., 2019; Hwang et al., 2023). Its surface accessibility and tumor enrichment in selected settings have also supported therapeutic development, including tumor-associated antigen vaccines, monoclonal antibodies, antibody-drug conjugates, bispecific T-cell redirectors, and radioimmunotherapy programs (Imai et al., 2008; Zhang et al., 2010; Root et al., 2016; Subbiah et al., 2020; Duca et al., 2022; Jung et al., 2024).

The CDH3 literature also contains clear tensions that preclude a uniform interpretation. In melanoma, forced or restored P-cadherin can strengthen intercellular adhesion and counter invasion, whereas in bladder carcinoma poor outcome may track with overexpression, loss, or cytoplasmic mislocalization depending on the model and specimen context (Van Marck et al., 2005; Mandeville et al., 2008; Jacobs et al., 2010; 2011). In hepatocellular carcinoma, both downregulation-associated tumorigenicity and KLF4-mediated CDH3 upregulation with growth suppression have been reported, underscoring how lineage-specific adhesion wiring alters biological output (Bauer et al., 2014; Li et al., 2019). Even within breast cancer, the consequence of P-cadherin expression depends strongly on whether E-cadherin is absent, preserved, or partially switched, and on whether CDH3 marks a basal-like compartment, a leader-cell subset, or a focal microenvironment-induced re-cohesion state (Ribeiro et al., 2013; Christgen et al., 2020; Hwang et al., 2023; Gronewold et al., 2024). These findings indicate that CDH3 function is shaped by context, localization, and interacting adhesion networks.

For a translational review, this heterogeneity matters because biomarker usefulness and druggability both depend on identifying the correct disease state rather than simply measuring bulk gene abundance. A high CDH3 transcript in one tumor may reflect a biologically invasive basal-like program, whereas in another it may indicate partial epithelial restoration or coexist with other cadherins that fundamentally reshape function. Likewise, therapeutic targeting requires more than evidence of expression. It requires an understanding of membrane accessibility, normal-tissue distribution, intratumoral heterogeneity, the interaction between CDH3 and survival signaling, and the extent to which CDH3-high states remain stable under treatment pressure.

This review addresses CDH3 as a context-governed adhesion-signaling node in human tumors. Rather than cataloguing every report, it synthesizes the most informative evidence across molecular regulation, tumor biology, cancer-type heterogeneity, biomarker development, and therapeutic translation. The core questions are how CDH3 is regulated across cancers, how it drives or restrains specific malignant phenotypes, why its role reverses in certain cellular backgrounds, and what obstacles must be overcome before CDH3-guided diagnostics or therapies become clinically actionable.

## Main text

### Molecular basis and physiological functions of CDH3/P-cadherin

CDH3 encodes P-cadherin, a classical type I cadherin located on chromosome 16q22.1 in close proximity to CDH1, the gene encoding E-cadherin. Like other classical cadherins, P-cadherin comprises five extracellular cadherin repeats, a single transmembrane segment, and a cytoplasmic tail that binds p120-catenin and β-catenin, thereby linking intercellular junctions to α-catenin-associated actin organization. This architecture allows P-cadherin to function as both an adhesive component and a signaling platform that couples cell-cell contact to polarity, tension, migration, and survival outputs. Biophysical work on human P-cadherin demonstrates calcium-dependent self-adhesion with defined structural and thermodynamic constraints, providing a mechanistic basis for its role in tissue cohesion and its potential to modulate collective behavior when dysregulated (Kudo et al., 2012).

In normal tissues, P-cadherin is enriched in basal or progenitor-associated epithelial compartments rather than uniformly distributed throughout mature epithelium. It is found in the basal layer of stratified epithelia, myoepithelial cells of the breast, hair follicles, placenta, and other sites characterized by turnover, repair, or specialized boundary organization (Albergaria et al., 2011). This spatial pattern suggests that CDH3 is linked to tissue renewal, compartmental integrity, and lineage demarcation. In the breast, P-cadherin is integral to normal mammary development and basal compartment maintenance, which helps explain why its re-expression in tumors often correlates with basal-like phenotypes and stem-associated features rather than simply reflecting generic dedifferentiation (Albergaria et al., 2011).

The relationship between P-cadherin and E-cadherin is especially important. They are related classical cadherins, but they are not interchangeable. E-cadherin is more closely associated with mature epithelial stability, whereas P-cadherin is linked to basal identity, epithelial plasticity, and boundary function. In tumors, the relationship between them is not a simple binary switch. Some cancers display a partial transition from E-cadherin to P-cadherin, while others maintain both molecules, with functional consequences depending on relative abundance and junctional organization. In breast cancer models, the pro-invasive function of P-cadherin depends strongly on the cellular E-cadherin context, showing that cadherin network composition is more informative than single-gene status alone (Ribeiro et al., 2013). In pancreatic ductal adenocarcinoma, CDH1 and CDH3 can cooperate rather than oppose one another, indicating that P-cadherin may complement rather than merely replace epithelial adhesion programs in certain aggressive tumors (Siret et al., 2018).

Normal physiological roles help explain later tumor phenotypes. Cadherins are central to epithelial sheet dynamics, tissue repair, and the transmission of mechanical information across cells. P-cadherin is therefore positioned to influence adhesion strength, collective epithelial movement, basal-compartment maintenance, and responses to injury or morphogenetic stress. This broader physiological framing is important because many malignant CDH3-associated behaviors, including collective invasion, leader-cell polarization, and microenvironmental coupling, resemble exaggerated versions of regulated tissue programs rather than wholly novel cancer-specific processes.

The normal biology of CDH3 supports two points. P-cadherin is well positioned to influence both adhesion and signaling because it couples directly to catenin complexes and actin-associated mechanics. In addition, its enrichment in basal, reparative, or developmentally plastic epithelial states suggests that tumor reactivation of CDH3 often reflects reuse of lineage-associated programs. This framework helps explain why CDH3 can promote aggressive behavior in many epithelial tumors while exerting restraining effects in selected settings.

### Dysregulated expression, epigenetic control, and post-transcriptional regulation in cancer

Across human tumors, CDH3 dysregulation most commonly appears as overexpression, but the biological meaning of “abnormal” CDH3 extends beyond abundance alone. In invasive breast carcinoma, P-cadherin overexpression is strongly associated with high histological grade, estrogen receptor negativity, high proliferation, and poor clinical outcome, establishing one of the earliest and most influential clinicopathological links between CDH3 and aggressive disease (Paredes et al., 2005). Subsequent large tissue microarray and meta-analytic studies confirmed that P-cadherin is a reproducible adverse biomarker in breast cancer, particularly within basal-like or high-risk subgroups (Turashvili et al., 2011; Sridhar et al., 2020; Qi et al., 2022). Similar associations have been reported in non-small cell lung cancer, colorectal cancer, pancreatic cancer, biliary malignancies, tubo-ovarian high-grade serous carcinoma, and glioblastoma, supporting the view that CDH3 frequently marks clinically aggressive epithelial tumors (Sakamoto et al., 2015; Kumara et al., 2017; Imai et al., 2018; Martins et al., 2022; Wang et al., 2022; Canário et al., 2025).

Some cancers highlight the limits of expression-only interpretations. In bladder carcinoma, adverse prognosis can be associated with CDH3 loss, non-restricted distribution, or cytoplasmic localization rather than simple overexpression, indicating that membrane organization and spatial restriction matter biologically (Mandeville et al., 2008). In melanoma, restoration of P-cadherin can strengthen adhesion and suppress invasion, whereas in renal cancer reduced expression and altered DNA methylation have been linked to aggressive behavior (Van Marck et al., 2005; Faraj Tabrizi et al., 2023). Hepatocellular carcinoma provides another contradictory example, with reports suggesting that loss of P-cadherin may increase tumorigenicity while KLF4-mediated CDH3 upregulation can restrain growth and migration (Bauer et al., 2014; Li et al., 2019). These observations indicate that CDH3 dysregulation should be understood through three axes: level, localization, and cellular context.

Epigenetic regulation is one of the most consistent mechanisms underlying CDH3 deregulation. In breast cancer, Paredes and colleagues showed that CDH3 overexpression is associated with promoter hypomethylation and that demethylating treatment can increase CDH3 expression, establishing promoter methylation status as a direct regulatory layer (Paredes et al., 2005). Similar evidence of CDH3 demethylation has been reported in advanced gastric carcinoma, reinforcing the idea that epigenetic derepression contributes to CDH3 activation beyond the breast setting (Hibi et al., 2009). In intrahepatic cholangiocarcinoma and pancreatic cancer, integrated transcriptomic and methylation analyses likewise support a link between high CDH3 expression and epigenetic activation during tumor progression (Sakamoto et al., 2015; Peng et al., 2021). At the other end of the spectrum, aggressive renal cell carcinoma has been associated with altered CDH3 DNA methylation and reduced expression, emphasizing that epigenetic change does not have a fixed phenotypic direction across lineages (Faraj Tabrizi et al., 2023).

Beyond methylation, higher-order chromatin organization also shapes CDH3 regulation. In gastric cancer, 3D chromatin architecture rewiring at the CDH3/CDH1 loci accompanies an E-cadherin-to-P-cadherin expression switch, shifting the discussion from single-promoter events to locus-scale topological reprogramming (São José et al., 2023). In breast cancer, endocrine pressure can alter chromatin accessibility: the estrogen receptor degrader ICI 182,780 induces P-cadherin overexpression through promoter-level chromatin remodeling and a role for C/EBP in CDH3 activation (Albergaria et al., 2010). Together with the observation that BRCA1 transcriptionally regulates genes associated with the basal-like phenotype, including programs linked to CDH3, these findings place CDH3 within broader lineage and therapy-associated epigenomic reprogramming rather than isolated gene-specific control (Gorski et al., 2010).

Transcription-factor and lineage programs further influence CDH3 abundance. C/EBPβ isoforms directly regulate the pro-invasive CDH3 gene in breast cancer cells, providing a mechanistic bridge between transcriptional state and invasive phenotype (Albergaria et al., 2013). In ovarian cancer, HOXA9 induces P-cadherin and facilitates both homotypic tumor aggregation and heterotypic tumor-mesothelium interactions, indicating that developmental transcriptional programs can repurpose CDH3 to support dissemination (Ko and Naora, 2014). This type of regulation helps explain why CDH3 expression often clusters with specific lineage states rather than functioning as a random marker across all tumors.

Noncoding RNA networks are now a major part of the CDH3 regulatory landscape. In esophageal cancer, the lncRNA ADAMTS9-AS2 suppresses progression by recruiting DNA methyltransferases to the CDH3 promoter, thereby reducing CDH3 expression and revealing one route by which noncoding RNAs can epigenetically silence a CDH3-driven malignant axis (Liu et al., 2020). In lung cancer, hypoxia-enriched exosomal lncRNA FGD5-AS1 promotes proliferation and invasion by sponging miR-1179 and releasing CDH3 from repression, exemplifying a microenvironment-dependent competing endogenous RNA pathway centered on CDH3 (Yang et al., 2025a). CircRNA-mediated regulation has also been reported: circ_0023179 modulates proliferation, apoptosis, and epithelial-mesenchymal transition in non-small cell lung cancer through a miR-615-5p/CDH3 axis (Guo et al., 2024). In gastrointestinal tumors, miR-665 and miR-133a suppress malignant phenotypes by directly targeting CDH3 in gastric and colorectal cancer, respectively, reinforcing the notion that CDH3 frequently serves as a convergent effector of tumor-suppressive miRNA programs (Fang et al., 2021; Sharma et al., 2023). Antisense regulation can also reverse the usual oncogenic pattern: in melanoma, CDH3-AS1 enhances P-cadherin translation and acts as a tumor suppressor, underscoring the context dependence of CDH3 biology even at the RNA-regulatory level (Chadourne et al., 2025).

Stress responses and protein homeostasis add another layer. Oxidative stress can increase CDH3 transcription in lung cancer cells through OGG1-mediated enhancement of SP1 binding, placing CDH3 within damage-responsive transcriptional circuitry (Ma et al., 2025b). Post-translationally, USP8-mediated stabilization of CDH3 has been linked to ferroptosis regulation and malignant progression in lung adenocarcinoma, indicating that CDH3 protein abundance is shaped by turnover control as well as transcriptional events (Qv et al., 2025).

**FIGURE 1:**
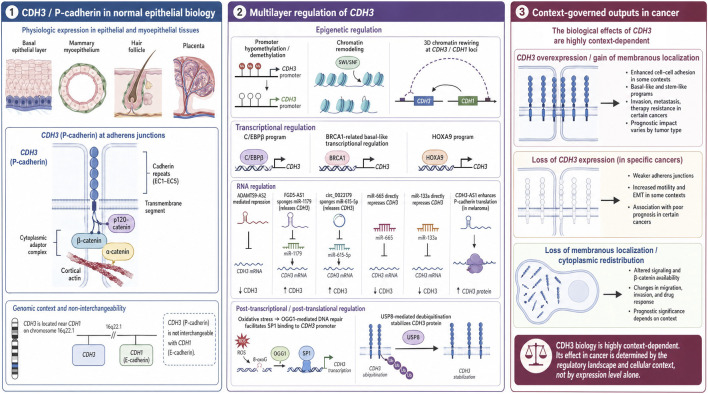
CDH3/P-cadherin biology and multilayer regulation in cancer. P-cadherin is a classical cadherin enriched in basal or progenitor-associated epithelial compartments and linked to catenin-actin complexes at cell-cell junctions. In cancer, CDH3 expression is shaped by promoter methylation status, chromatin remodeling, 3D locus rewiring, lineage-specific transcriptional programs, noncoding RNA networks, oxidative stress-responsive transcription, and USP8-mediated protein stabilization. The biological consequence depends on abundance, localization, and tissue context rather than expression level alone.

The regulatory literature supports three conclusions. CDH3 dysregulation is recurrent across cancers, but it is not synonymous with simple overexpression. Promoter methylation, chromatin remodeling, lineage transcription factors, and noncoding RNAs form an integrated multilayer system that can either activate or suppress CDH3 depending on context. Apparent conflicts in phenotype often become more interpretable when membrane localization, cadherin background, and the regulatory state that produced CDH3 expression are considered together (Figure 1).

## Tumor-biological mechanisms driven by CDH3

### Proliferation, survival, and receptor signaling amplification

CDH3 contributes to malignancy by modulating adhesion and amplifying survival-associated signaling. In oral dysplastic and malignant keratinocytes, P-cadherin potentiates ligand-dependent epidermal growth factor receptor (EGFR) and insulin-like growth factor 1 receptor (IGF-1R) signaling, prolonging downstream pathway activation and linking membrane adhesion status to mitogenic output (Lysne et al., 2014). In ovarian cancer, P-cadherin induction downstream of HOXA9 strengthens cell-cell aggregation and tumor-peritoneum interaction, thereby enhancing dissemination and creating a platform for prosurvival tumor-mesothelium communication (Ko and Naora, 2014; Usui et al., 2014). Taken together, these studies suggest that CDH3 often behaves more like a membrane organizer or signal amplifier than a purely upstream oncogenic initiator.

Evidence from premalignant and basal-like breast models extends this idea. Activation of an actin signaling pathway by P-cadherin is essential for transformation in premalignant mammary epithelial cells, indicating that CDH3 can participate very early in malignant conversion rather than only in late invasion (Faria et al., 2023). In basal-like breast cancer cells, SRC inhibition blocks P-cadherin-mediated signaling and function, demonstrating that therapeutically relevant downstream dependencies can be created by the CDH3 state itself (Ribeiro et al., 2018). In lung adenocarcinoma, CDH3 has also been linked to resistance mechanisms through ferroptosis regulation and oxidative stress-associated transcriptional reinforcement, broadening its functional reach from classical proliferation toward stress-adaptive survival (Ma et al., 2025b; Qv et al., 2025). More specifically, the USP8-CDH3 axis provides a plausible protein-stability route through which lung adenocarcinoma cells may dampen ferroptotic vulnerability. USP8-mediated deubiquitination stabilizes CDH3 protein, and increased CDH3 then supports malignant phenotypes while suppressing ferroptosis-associated cell death (Qv et al., 2025). Because ferroptosis is driven by iron-dependent lipid peroxidation and restrained by antioxidant systems such as the GSH/GPX4 axis, this finding links CDH3 to a stress-survival program rather than to adhesion alone. In parallel, oxidative stress can transcriptionally reinforce CDH3 expression through OGG1-mediated enhancement of SP1 binding at the CDH3 promoter, suggesting a feed-forward context in which oxidative damage increases CDH3 abundance while stabilized CDH3 helps tumor cells tolerate ferroptosis-inducing pressure (Ma et al., 2025b; Qv et al., 2025). At present, the evidence supports this USP8-CDH3-ferroptosis cascade at the pathway level; whether CDH3 directly regulates GPX4, glutathione metabolism, or lipid-remodeling enzymes remains to be defined.

Current evidence supports a model in which CDH3-high states potentiate receptor and stress-response pathways in a context-dependent manner. What remains less resolved is whether P-cadherin acts as a dominant upstream organizer across tumors or more often as a reinforcing component that stabilizes pre-existing aggressive signaling programs. This distinction matters therapeutically because combination strategies may outperform direct CDH3 targeting in tumors where P-cadherin mainly serves as a pathway amplifier.

### Epithelial-mesenchymal transition, migration, invasion, and collective movement

Migration and invasion are the best-characterized consequences of CDH3 dysregulation, but the underlying mechanisms are diverse. In colon cancer, P-cadherin promotes liver metastasis and poor prognosis, partly through cooperation with Wnt signaling and changes consistent with a cadherin-switch phenotype (Sun et al., 2011). In cholangiocarcinoma and prostate cancer models, CDH3 affects cell motility through pathways involving p120-catenin and non-E-cadherin cadherin networks, further indicating that CDH3-mediated invasion is not reducible to a single canonical epithelial-mesenchymal transition route (Baek et al., 2010; Kümper and Ridley, 2010). In lung adenocarcinoma, recent work links CDH3 directly to epithelial-mesenchymal transition progression, integrating prior clinicopathological evidence with a mechanistic pro-invasive framework (Gong et al., 2025).

An important advance in the field has been the recognition that CDH3 is central to collective migration and tissue mechanics. P-cadherin promotes collective migration by increasing Cdc42-mediated mechanical forces and coordinating Rho GTPase-dependent tension across cell groups (Plutoni et al., 2016b; 2016a). These findings reposition CDH3 from a molecule that merely permits movement to one that helps organize how cells move together. Consistent with this, P-cadherin-induced decorin secretion is required for collagen fiber alignment and directional collective migration, demonstrating that CDH3 can reshape the extracellular matrix to generate paths for coordinated invasion (Le Borgne-Rochet et al., 2019). In breast tumor leader cells, a Cdh3-β-catenin-laminin signaling axis controls leader-cell polarization and directional collective migration, indicating that CDH3 can specify a specialized invasive subpopulation with disproportionate influence on tumor movement (Hwang et al., 2023).

This collective-migration literature is especially important for translational interpretation because it suggests that CDH3 marks more than a bulk increase in motility. It may define a mechanically competent, invasion-organizing cell state. Such a state is likely to be underrepresented by average transcript abundance alone and may require spatial or functional assays to detect clinically.

### Stemness, metabolic adaptation, and resistance to detachment-induced death

CDH3 also contributes to aggressive tumor behavior by supporting plastic and stress-tolerant cell states. In basal-like breast cancer, P-cadherin is coexpressed with CD44 and CD49f and mediates stem-cell properties, linking it to tumor-initiating potential and phenotypic plasticity (Vieira et al., 2012). The same tumor context reveals a connection between P-cadherin and a glycolytic, acid-resistant phenotype, suggesting that CDH3-high populations are metabolically equipped for hostile microenvironments (Sousa et al., 2014). This is complemented by evidence that P-cadherin induces anoikis resistance in matrix-detached breast cancer cells by promoting the pentose phosphate pathway and decreasing oxidative stress, thereby supporting survival during detachment and dissemination (Sousa et al., 2020).

Ovarian cancer provides a mechanistically distinct but conceptually related example. P-cadherin can mechanoactivate metabolic coupling between tumor cells and mesothelial surfaces to promote metastasis, showing that CDH3 can support dissemination through cohesion and aggregation as well as through metabolic symbiosis with the microenvironment (Ma et al., 2025a). These data broaden the CDH3 field beyond invasion to include metabolic support programs that may help explain why CDH3-high tumors often resist treatment and colonize secondary niches more efficiently.

The stemness and metabolism literature remains more concentrated in specific model systems than the invasion literature, but the overall pattern is coherent: CDH3 is repeatedly associated with cell states that are invasive, stress-tolerant, and able to survive detachment or hostile environments. This argues that CDH3 is particularly relevant to the metastatic cascade rather than merely to primary tumor growth.

### Hypoxia, extracellular communication, and ceRNA networks

A final mechanistic theme is the integration of CDH3 into microenvironmental communication networks. Hypoxia is a common feature of solid tumors, and recent data in lung cancer indicate that hypoxia can promote secretion of exosomes enriched in lncRNA FGD5-AS1, which in turn relieves miR-1179-mediated repression and supports CDH3-dependent malignant phenotypes (Yang et al., 2025a). In this setting, CDH3 is not merely a downstream gene; it is the node through which hypoxia, exosomal communication, and noncoding RNA control converge.

This theme aligns with the broader observation that P-cadherin is closely involved in cell-cell and cell-matrix information transfer. The literature on collective movement, tumor-mesothelium interaction, and extracellular matrix remodeling suggests that CDH3-high tumors efficiently convert contact-dependent cues into coordinated invasive behavior. In this context, CDH3 appears to function as a central component of multicellular tumor organization rather than as an isolated molecular marker.

**FIGURE 2:**
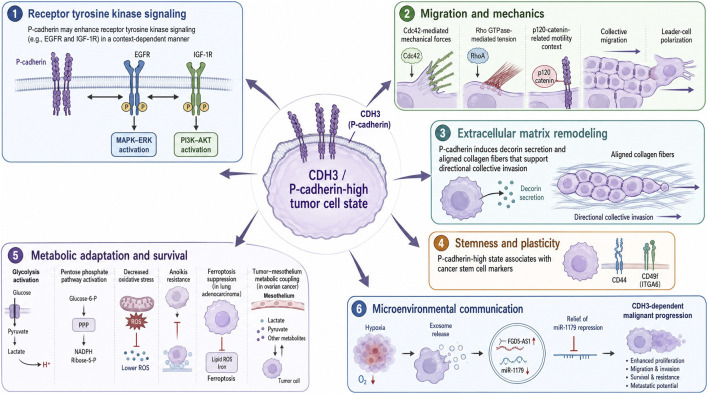
Tumor-biological mechanisms associated with CDH3-high states. CDH3 can amplify receptor signaling, support collective migration and extracellular-matrix remodeling, reinforce leader-cell behavior, associate with stem-like traits, promote metabolic and detachment-resistant survival states, and integrate hypoxia-exosome-ceRNA signaling. The downstream circuitry differs across tumor types, but the recurring theme is that CDH3 links adhesion status to invasive and stress-adaptive programs.

The mechanistic record is strongest for three functions: amplification of signaling, orchestration of collective invasion, and support of plastic stress-resistant states. Evidence for each comes from multiple cancer contexts, although the downstream wiring differs. Translational strategies based on CDH3 will therefore require context-specific biomarker frameworks rather than a single pan-cancer rule (Figure 2).

## Context-dependent roles across major tumor types

### Determinants of CDH3 functional polarity

The divergent behavior of CDH3 can be organized around three major determinants. The first is tissue type and cell of origin. In basal-like or ductal epithelial programs, CDH3 often marks plastic, invasive, or aggregation-prone states, whereas in melanoma, hepatocellular carcinoma, and selected bladder or oral cancer contexts, restoration of P-cadherin-mediated adhesion can restrain migration or tumorigenicity (Van Marck et al., 2005; Mandeville et al., 2008; Bauer et al., 2011; 2014; Li et al., 2019). The second determinant is the E-cadherin background. CDH3 does not simply replace CDH1; instead, its functional output depends on whether E-cadherin is lost, retained, switched, or locally re-expressed, as shown most clearly in breast and pancreatic models (Ribeiro et al., 2013; Siret et al., 2018; Christgen et al., 2020; Gronewold et al., 2024). The third determinant is subcellular localization. Membrane-localized P-cadherin can support cohesive signaling, collective migration, or adhesive restraint depending on lineage, whereas cytoplasmic redistribution or loss of spatial restriction may indicate junctional dysfunction and adverse biology, particularly in bladder carcinoma and melanoma (Mandeville et al., 2008; Van Marck et al., 2011). Microenvironmental cues further modulate these determinants by inducing focal CDH3 expression, leader-cell states, or tumor-host adhesive interactions (Hwang et al., 2023; Gronewold et al., 2024; Ma et al., 2025a).

This framework helps explain why CDH3 is predominantly interpreted as pro-tumor in many epithelial malignancies but as suppressive or bidirectional in selected lineages. Hepatocellular carcinoma is a particularly important exception: reduced P-cadherin expression has been associated with increased tumorigenicity, whereas KLF4-mediated CDH3 upregulation suppresses hepatoma cell growth and migration through GSK-3β-related signaling (Bauer et al., 2014; Li et al., 2019). Thus, CDH3 polarity should not be inferred from expression level alone; it requires joint interpretation of lineage, cadherin-network state, membrane localization, and the signaling pathway in which P-cadherin is embedded.

The strength of evidence is uneven across tumor types and should be interpreted hierarchically. Breast cancer represents the most mature setting because it combines large clinicopathological cohorts, meta-analytic evidence, mechanistic models, and therapeutic rationale. Lung cancer, colorectal cancer, pancreatobiliary tumors, ovarian cancer, and glioblastoma provide increasingly informative but more heterogeneous evidence, often combining retrospective cohorts, computational analyses, and selected functional studies. Hepatocellular carcinoma, melanoma, bladder carcinoma, oral squamous cell carcinoma, and renal cancer are especially valuable as counterexamples or lineage-specific settings, but their evidence should not be weighted as equivalent to the breast cancer literature. This hierarchy is summarized in Table 1.

**TABLE 1:** Context-specific interpretation and translational maturity of CDH3 across major tumor types.

Tumor setting	Predominant CDH3 pattern	Main functional interpretation	Biomarker evidence	Therapeutic targeting stage	Key citation
Breast cancer	Frequently overexpressed, especially in basal-like or high-risk disease	Pro-invasive, stem-like, glycolytic, collective-migration-associated, but strongly dependent on E-cadherin context	Most mature: large cohorts, tissue microarrays, meta-analyses, and mechanistic studies	Strong therapeutic rationale; CDH3-directed agents are relevant but not established as standard care	Paredes et al. (2005), Turashvili et al. (2011), Ribeiro et al. (2013), Sridhar et al. (2020), Qi et al. (2022), Hwang et al. (2023)
Lung cancer	Frequently overexpressed in NSCLC/LUAD; soluble and transcript-based signals also reported	Pro-tumor signaling, EMT, oxidative-stress response, ferroptosis suppression, ceRNA-linked regulation	Emerging-to-moderate: prognostic cohorts, omics models, liquid-biopsy studies, and recent functional work	Preclinical and biomarker-driven rationale; no established disease-specific CDH3 therapy	Imai et al. (2018), Hsiao et al. (2020), Ma and Hu (2024), Ma et al. (2025b), Qv et al. (2025), Yang et al. (2025a)
Gastric and colorectal cancer	Often overexpressed or epigenetically activated	Dissemination, cadherin-switch biology, metastasis, and serum/diagnostic-marker potential	Moderate: epigenetic studies, tissue/serum biomarker reports, and functional evidence	Tumor-associated antigen rationale and preclinical targeting relevance	Hibi et al. (2009), Sun et al. (2011), Kumara et al. (2017), Fang et al. (2021), Song et al. (2024), Fu et al. (2025)
Pancreatic and biliary malignancies	Frequently overexpressed in selected pancreaticobiliary contexts	Aggressive epithelial cohesion, migration, diagnostic aid, and targetable surface antigenicity	Moderate: tissue studies, biliary brush cytology, methylation/transcriptomic evidence	Strong preclinical/early translational target rationale; clinical benefit remains unproven	Imai et al. (2008), Baek et al. (2010), Sakamoto et al. (2015), Kim et al. (2016), Siret et al. (2018)
Ovarian and tubo-ovarian serous carcinoma	Overexpressed in dissemination-prone and aggressive serous settings	Aggregation, tumor-peritoneum adhesion, mesothelial interaction, and metabolic coupling	Emerging-to-moderate: functional models and recent pathology evidence	Preclinical target rationale, especially for blocking adhesion/metabolic coupling	Ko and Naora (2014), Usui et al. (2014), Ma et al. (2025a)
Hepatocellular carcinoma	Often reduced or functionally restored in suppressive contexts	Mainly tumor-suppressive or bidirectional; low expression may promote tumorigenicity, whereas KLF4-mediated upregulation suppresses growth and migration	Limited but mechanistically informative	No established CDH3-targeted therapeutic program	Bauer et al. (2014), Li et al. (2019)
Melanoma	Restored or membrane-localized P-cadherin can be suppressive; cytoplasmic redistribution may reflect dysfunction	Adhesive restraint and invasion suppression	Mechanistic counterexample evidence	No established therapeutic targeting role	Van Marck et al. (2005), Jacobs et al. (2010), 2011; Chadourne et al. (2025)
Bladder carcinoma	Loss, non-restricted distribution, or cytoplasmic localization may be clinically relevant	Localization-dependent migration/prognostic effect rather than simple overexpression	Moderate pathology and functional evidence	No established CDH3-targeted therapeutic program	Mandeville et al. (2008), Van Marck et al. (2011)
Glioblastoma and selected other tumors	Often reported as overexpressed in specific datasets	Oncogenic biomarker or context-specific modifier	Emerging: cohort and functional studies	Conceptual/preclinical relevance; clinical translation not established	Martins et al. (2022), Wang et al. (2022), Liu et al. (2025), Pavlova et al. (2025)

### Breast cancer: the most mature evidence base

Breast cancer provides the clearest example of CDH3 as an aggressive yet context-dependent marker. Early clinicopathological studies established a robust link between P-cadherin overexpression, high grade, high proliferation, estrogen receptor negativity, and poor outcome (Paredes et al., 2005). Large cohorts and meta-analyses later confirmed its prognostic significance, especially in basal-like disease (Turashvili et al., 2011; Sridhar et al., 2020; Qi et al., 2022). Mechanistically, breast cancer studies also offer the richest regulatory framework, spanning promoter hypomethylation, chromatin remodeling under endocrine pressure, C/EBPβ-mediated transcription, and BRCA1-associated basal-like reprogramming (Paredes et al., 2005; Albergaria et al., 2010; Albergaria et al., 2013; Gorski et al., 2010).

Importantly, breast cancer has also clarified why CDH3 cannot be interpreted outside its cadherin context. P-cadherin function is strongly dependent on E-cadherin status, and different breast cancer subtypes can display coexistence, switching, or focal re-expression of these cadherins with distinct consequences (Ribeiro et al., 2013; Christgen et al., 2020; Gronewold et al., 2024). At the invasive front, a leader-cell subset uses a Cdh3-β-catenin-laminin axis to control collective migration, indicating that CDH3 may identify the most motility-competent fraction rather than merely the largest one (Hwang et al., 2023). Breast cancer therefore illustrates the full CDH3 spectrum: prognostic biomarker, mechanistic driver, lineage marker, and spatial organizer of invasion.

### Lung cancer: convergence of prognostic, mechanistic, and liquid-biopsy evidence

In lung cancer, CDH3 has progressed from an expression marker to a more mechanistically and translationally integrated feature. Early exon-array studies already implicated CDH3 among differentially expressed cancer-related genes in lung cancer (Xi et al., 2008). Subsequent clinical work showed that high P-cadherin expression correlates with poor prognosis in non-small cell lung cancer (Imai et al., 2018). More recent studies extend this by linking CDH3 to epithelial-mesenchymal transition, oxidative-stress signaling, ferroptosis control, ceRNA networks, and broader cadherin-family prognostic models (Gong et al., 2025; Ma et al., 2025b; Qv et al., 2025; Yang et al., 2025b).

Lung cancer is also notable for apparently discordant findings. Most reports support a pro-tumor role. One study found that lower CDH3 was associated with a better prognosis in lung adenocarcinoma and that CDH3 knockdown reduced proliferation and migration, a pattern still broadly consistent with a pro-tumor interpretation but sensitive to cohort composition and analytic framing (Ma and Hu, 2024). Soluble cadherin-3 has shown promise as an early monitoring marker for epidermal growth factor receptor tyrosine kinase inhibitor therapy, and a machine-learning-based Wnt-related diagnostic model placed CDH3 among robust lung adenocarcinoma diagnostic markers (Hsiao et al., 2020; Liu et al., 2026). Lung cancer therefore provides one of the clearest settings in which CDH3 biology intersects with clinically testable biomarker strategies.

### Hepatocellular carcinoma: a lineage-specific tumor-suppressive context

Hepatocellular carcinoma illustrates why CDH3 should not be treated as uniformly oncogenic across epithelial tumors. In contrast to breast, lung, pancreaticobiliary, and ovarian settings, available HCC evidence suggests a predominantly suppressive or bidirectional pattern. Downregulation of P-cadherin expression has been reported to increase tumorigenicity, implying that loss of this adhesion component may release hepatocellular tumor cells from growth- or architecture-restraining control (Bauer et al., 2014). Conversely, KLF4-mediated CDH3 upregulation suppresses human hepatoma cell growth and migration, supporting the concept that higher CDH3 expression can restrain malignant behavior in this lineage (Li et al., 2019).

Mechanistically, this HCC phenotype appears to intersect with GSK-3β and Wnt/β-catenin signaling. KLF4-induced CDH3 expression has been linked to GSK-3β-related suppression of growth and migration, which is biologically relevant because GSK-3β can restrain β-catenin-dependent transcriptional output within the Wnt pathway (Li et al., 2019). Thus, in HCC, CDH3 may reinforce adhesive organization and signaling restraint rather than promote invasion. This liver-specific interpretation helps explain the bidirectional pattern: low CDH3 may favor tumorigenesis, whereas restored or high CDH3 may suppress malignancy through KLF4-CDH3-GSK-3β/Wnt-β-catenin-associated control.

### Gastric, colorectal, pancreatic, and biliary malignancies: epigenetic activation and dissemination

Gastrointestinal and pancreatobiliary tumors collectively support a predominantly pro-tumor interpretation of CDH3, although the mechanisms vary by site. In gastric cancer, frequent CDH3 demethylation and 3D chromatin rewiring support epigenetic activation, while miR-665-mediated repression of CDH3 indicates continued selection pressure to restrain its expression in malignant cells (Hibi et al., 2009; Fang et al., 2021; São José et al., 2023). In colorectal cancer, P-cadherin overexpression has been associated with metastasis and poor prognosis, and circulating or serum-associated CDH3 signals have shown potential for disease monitoring, particularly in distant metastasis (Sun et al., 2011; Kumara et al., 2017; Song et al., 2024). Bioinformatic and functional work further suggests that CDH3 participates in broader colorectal cancer progression programs and may intersect with immunotherapy-relevant networks (Sharma et al., 2023; Fu et al., 2025; Saleh et al., 2025).

Pancreatic and biliary tumors add a strong translational layer. CDH3 was originally identified as a tumor-associated antigen in pancreatic, gastric, and colorectal cancers, supporting the notion that these tumors can expose CDH3-derived immunogenic targets (Imai et al., 2008). In pancreatic ductal adenocarcinoma, cooperation between cadherin-1 and cadherin-3 determines aggressiveness, emphasizing that epithelial cohesion and invasion can be co-optimized rather than mutually exclusive (Siret et al., 2018). In cholangiocarcinoma and biliary tract malignancy, overexpression of P-cadherin is tied to progression, diagnosis, and migration control, and CDH3 mRNA has shown utility in biliary brush cytology specimens (Baek et al., 2010; Riener et al., 2010; Kim et al., 2016). These cancers therefore position CDH3 at the intersection of dissemination biology, diagnostic aid, and targetable surface antigenicity.

### Ovarian cancer and tubo-ovarian serous neoplasia: aggregation, mesothelial interaction, and metabolic coupling

In ovarian cancer, the most coherent theme is that CDH3 promotes spread by facilitating multicellular organization and tumor-host interaction. HOXA9-driven P-cadherin expression enhances homotypic aggregation and heterotypic interactions that favor peritoneal dissemination, while functional studies show that P-cadherin promotes ovarian cancer spread through tumor cell aggregation and tumor-peritoneum adhesion (Ko and Naora, 2014; Usui et al., 2014). More recent work indicates that P-cadherin can mechanoactivate metabolic coupling between tumor cells and mesothelial cells, broadening dissemination biology into the metabolic domain (Ma et al., 2025a). In tubo-ovarian high-grade serous carcinoma, P-cadherin overexpression is associated with early transformation of the fallopian tube epithelium and tumor aggressiveness, suggesting that CDH3 may participate in both early serous carcinogenesis and metastatic efficiency (Canário et al., 2025).

### Melanoma, bladder cancer, and other informative counterexamples

The strongest challenge to a universal oncogenic model comes from melanoma. In human melanoma models, P-cadherin can promote cell-cell adhesion and counter invasion, counteract myosin II-B function, and reduce tumor growth and responsiveness to growth factors *in vivo* (Van Marck et al., 2005; Jacobs et al., 2010; 2011). The more recent discovery that CDH3-AS1 enhances P-cadherin translation and behaves as a tumor suppressor in melanoma reinforces the idea that CDH3 can stabilize a less invasive adhesive state in certain neural crest-derived contexts (Chadourne et al., 2025).

Bladder carcinoma provides a related example: poor prognosis may reflect CDH3 loss, cytoplasmic redistribution, or loss of spatial restriction, not just overexpression (Mandeville et al., 2008; Van Marck et al., 2011). This pattern suggests that localization-aware pathology may be more informative than bulk expression quantification in some tumor types. Other contexts also complicate the picture. In glioblastoma, CDH3 behaves as an oncogenic biomarker with prognostic value (Martins et al., 2022). In tongue squamous cell carcinoma, P-cadherin expression correlates with poor prognosis and local immune status. In oral squamous cell carcinoma, Slit-2-facilitated interaction between P-cadherin and Robo-3 inhibits migration, showing that even within head and neck malignancies CDH3 output can vary (Bauer et al., 2011; Wang et al., 2022). In prostate cancer, extracellular-vesicle transcriptomics and tissue studies support diagnostic and biological relevance, but the directionality is modulated by the broader cadherin environment (Royo et al., 2016; Ferreira et al., 2018).

**FIGURE 3:**
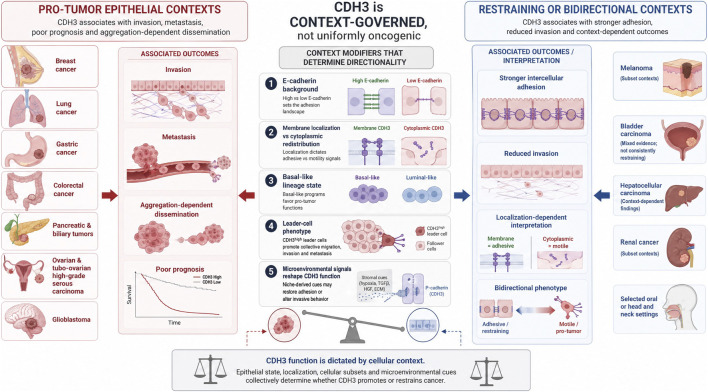
Context-dependent roles of CDH3 across tumor types. CDH3 is predominantly associated with aggressive behavior in many epithelial malignancies, especially when linked to basal-like states, collective invasion, or aggregation-dependent dissemination. In melanoma and selected bladder, liver, renal, and oral contexts, stronger adhesion, altered localization, or different cadherin wiring can shift CDH3 toward restraining or bidirectional phenotypes.

Across cancers, the heterogeneity data support one central conclusion: CDH3 is predominantly pro-tumor in many epithelial malignancies, especially when it participates in basal-like, invasive, or aggregation-dependent states. Yet suppressive or bidirectional effects emerge in tumors where stronger intercellular adhesion restrains invasion, where membrane localization is lost, or where cadherin network composition changes the functional meaning of CDH3 expression. Any translational use of CDH3 must therefore incorporate tumor type, cadherin context, and subcellular organization (Figure 3).

### Diagnostic, prognostic, and liquid-biopsy relevance

The biomarker potential of CDH3 has become one of the most clinically attractive aspects of the field. Tissue-based evidence is strongest in breast cancer, where P-cadherin consistently stratifies aggressive disease and adverse prognosis across early studies, large tissue microarrays, and meta-analyses (Paredes et al., 2005; Turashvili et al., 2011; Sridhar et al., 2020; Qi et al., 2022). Similar prognostic associations have been described in lung cancer, glioblastoma, tongue squamous cell carcinoma, and tubo-ovarian serous carcinoma, although the strength of evidence varies across cohorts and methodologies (Imai et al., 2018; Martins et al., 2022; Wang et al., 2022; Canário et al., 2025). In colorectal cancer and pancreatobiliary tumors, CDH3 appears useful both as a marker of biological aggressiveness and as part of multi-marker diagnostic frameworks (Riener et al., 2010; Sun et al., 2011; Kim et al., 2016).

A key translational advantage of CDH3 is that its value may increase when integrated with other markers rather than used in isolation. In lung adenocarcinoma, a Wnt-related diagnostic model incorporating CDH3 demonstrated strong cross-cohort performance, suggesting that CDH3 can contribute to clinically usable signatures even when it is not a standalone determinant (Liu et al., 2026). In biliary brush cytology, CDH3 mRNA improves diagnostic interpretation when combined with other malignant-stricture markers (Kim et al., 2016). In breast cancer, P-cadherin becomes especially informative when interpreted alongside estrogen receptor status, proliferation markers, and basal-like programs rather than as a generic positive/negative readout.

Liquid-biopsy and minimally invasive applications are increasingly plausible but remain unevenly mature. In prostate cancer, urine extracellular-vesicle transcriptomics identified altered CDH3 signals, supporting a noninvasive route for disease detection or characterization (Royo et al., 2016). In lung cancer, soluble cadherin-3 has been proposed as both a survival predictor and an early monitoring marker during EGFR tyrosine kinase inhibitor therapy, making CDH3 relevant for diagnosis as well as dynamic treatment assessment (Hsiao et al., 2020). In colorectal cancer, serum CDH3 has shown potential for monitoring distant metastasis, and this is complemented by exosome-related regulatory evidence in lung cancer that could eventually inform circulating RNA panels (Kumara et al., 2017; Song et al., 2024; Yang et al., 2025a).

Despite these encouraging data, important limitations remain. Most liquid-biopsy observations come from single-disease or modest-size cohorts, often without standardized assays or head-to-head comparison against established biomarkers. Furthermore, CDH3 biology raises a specific caution: membrane expression, soluble ectodomain release, extracellular-vesicle RNA abundance, and bulk tissue transcript levels may reflect different biological states. A high soluble CDH3 signal, for example, may indicate shedding, therapy-induced remodeling, or tumor burden, whereas high tissue membrane CDH3 may indicate a mechanically cohesive invasive phenotype. Translational development will therefore require careful analytical validation and biological interpretation rather than assuming that all CDH3-derived measurements are interchangeable.

Current evidence supports CDH3 as a biomarker family rather than a single biomarker. Tissue expression, soluble fragments, extracellular-vesicle transcripts, and integrated multigene panels offer complementary information. Further progress will depend on standardized assays, localization-aware pathology, and prospective validation in biomarker-guided clinical settings.

### Therapeutic targeting strategies and translational barriers

CDH3 is attractive therapeutically because it combines tumor-biological relevance with cell-surface accessibility, but the translational record must be separated into preclinical feasibility and genuine clinical progress. The earliest therapeutic rationale came from identification of CDH3 as a tumor-associated antigen capable of generating cytotoxic T-lymphocyte epitopes in pancreatic, gastric, and colorectal cancers (Imai et al., 2008). This established immunological targetability, but it did not by itself demonstrate clinical efficacy.

At the preclinical level, several CDH3-directed modalities have shown encouraging activity. PF-03732010, a fully human anti-P-cadherin monoclonal antibody, inhibited tumor growth and metastasis in experimental models, and gastric cancer studies suggested that antibody-mediated P-cadherin downregulation can reduce migration and tumor growth (Zhang et al., 2010; Park et al., 2012). PCA062, a P-cadherin-targeting antibody-drug conjugate, displayed potent activity in P-cadherin-expressing malignancy models (Sheng et al., 2021). PF-06671008, an anti-P-cadherin/anti-CD3 bispecific DART molecule, showed strong preclinical T-cell-redirecting activity (Root et al., 2016). Radioimmunotherapy and newer bispecific designs, including TRAILR2/CDH3 constructs, further support the feasibility of using CDH3 as a delivery or docking antigen (Yoshioka et al., 2012; Jung et al., 2024).

Clinical progress, however, remains early and should be interpreted cautiously. PCA062 entered a first-in-human phase I dose-escalation study, confirming clinical translation but not establishing broad therapeutic efficacy (Duca et al., 2022). PF-06671008 also progressed into phase I testing in advanced solid tumors, demonstrating that CDH3-directed T-cell engagement is clinically testable while leaving optimal patient selection and efficacy thresholds unresolved (Harding et al., 2022). FF-21101-based radioimmunotherapy programs have generated nonhuman primate dosimetry data and early clinical evaluation, but dosimetry, lesion heterogeneity, and organ exposure remain practical constraints (Subbiah et al., 2020; Funase et al., 2021; Ravizzini et al., 2023). Thus, CDH3 has advanced beyond theoretical target nomination, but it has not yet become an established therapeutic standard.

The central translational bottlenecks are target biology and therapeutic window. CDH3 expression is heterogeneous across tumor types, across patients within the same tumor type, and within individual tumors. Normal-tissue expression in basal epithelial compartments and placenta-associated biology narrows the margin for on-target/off-tumor toxicity. In addition, therapeutically relevant CDH3 is not equivalent to total mRNA or bulk protein abundance: membrane density, spatial continuity, internalization, shedding, and coexistence with other cadherins may determine whether a tumor is vulnerable to an antibody, ADC, CD3 bispecific, or radioimmunotherapy approach. These constraints explain why preclinical promise has not yet translated into routine clinical use.

The therapeutic lesson is therefore nuanced. CDH3 is a validated surface-accessible target with multiple workable platforms, but its clinical development will likely require biomarker-rich trial design, membrane-resolved companion diagnostics, rational combinations, and modality-specific thresholds for antigen density and heterogeneity. Future CDH3-targeted therapy should distinguish tumors in which CDH3 is merely expressed from tumors in which CDH3 is stable, accessible, and biologically actionable.

**FIGURE 4:**
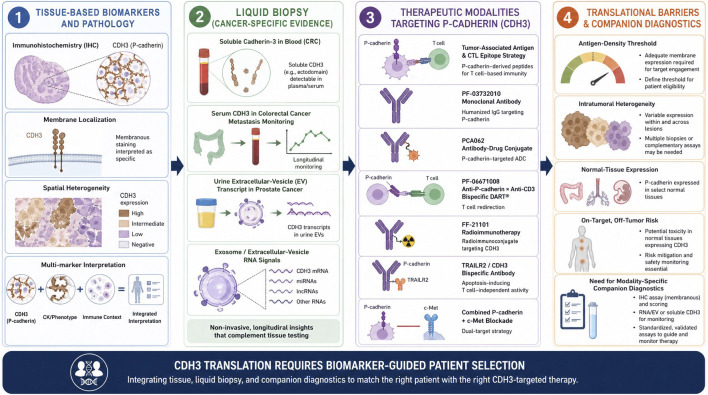
Biomarker and therapeutic translation landscape of CDH3. CDH3 can be assessed through tissue pathology, soluble and extracellular-vesicle-based measurements, and integrated diagnostic panels. Therapeutic development has advanced across antigen-based immunologic targeting, monoclonal antibodies, antibody-drug conjugates, bispecific T-cell engagement, radioimmunotherapy, and apoptosis-inducing bispecific constructs. Translation remains limited by heterogeneity, normal-tissue expression, and the need for modality-specific companion diagnostics.

A practical implication is that CDH3-targeted drug development and CDH3-directed biomarker development should no longer proceed as separate tracks. The assays used to define whether a tumor is “CDH3 positive” for pathology purposes may be insufficient to determine suitability for an ADC, a CD3 bispecific, or a radioimmunotherapy program. Each modality likely has a different threshold for antigen density, accessibility, internalization behavior, and heterogeneity tolerance. Future trials would benefit from paired tissue and liquid-biomarker strategies that combine membrane-resolved immunohistochemistry with soluble or extracellular-vesicle measurements. Such integrated companion diagnostics could help distinguish tumors with stable, therapeutically actionable CDH3 from those in which expression is focal, shed, treatment-induced, or biologically incidental (Figure 4).

### Challenges, controversies, and future directions

The greatest conceptual challenge in the CDH3 field is the coexistence of strong oncogenic evidence with clear counterexamples. This pattern likely reflects the biology of a molecule whose function depends on lineage, adhesion context, and membrane state. In epithelial tumors such as basal-like breast cancer, pancreatic ductal adenocarcinoma, cholangiocarcinoma, ovarian cancer, and subsets of lung cancer, CDH3 often participates in aggressive multicellular programs involving invasion, aggregation, or stress resistance. In melanoma and selected bladder or liver contexts, stronger or restored adhesion through P-cadherin can instead oppose invasion or tumorigenicity (Van Marck et al., 2005; Mandeville et al., 2008; Jacobs et al., 2011; Bauer et al., 2014; Li et al., 2019). Future work should address this duality mechanistically rather than averaging it away statistically.

A second challenge is the gap between bulk expression and functional state. Many studies quantify CDH3 mRNA or total protein, but far fewer resolve whether P-cadherin is membrane-bound, cytoplasmic, shed, leader-cell enriched, or embedded in a specific cadherin network. Yet these distinctions likely determine whether CDH3 promotes cohesion, collective invasion, or functional restraint. Localization-aware pathology, spatial transcriptomics, and high-resolution imaging of cell-state neighborhoods will therefore be essential.

Third, the field still leans heavily on *in vitro* systems and xenografts. These models have been invaluable for defining EGFR/IGF-1R signaling, Rho GTPase-mediated mechanics, metabolic adaptation, and antibody-based targeting, but they incompletely capture immune interactions, stromal constraints, and dynamic lineage transitions. Findings in leader-cell biology, tumor-mesothelium metabolic coupling, and microenvironment-induced focal P-cadherin restoration indicate that organoids, co-culture systems, and spatially resolved patient-derived models will be necessary to define the clinically relevant CDH3 state more precisely (Hwang et al., 2023; Gronewold et al., 2024; Ma et al., 2025b).

Fourth, translational progress requires more precise patient stratification. A future companion diagnostic for CDH3-targeted therapy will probably need to integrate tumor type, membrane antigen density, intratumoral heterogeneity, soluble or extracellular-vesicle readouts, and co-dependencies such as EGFR, c-Met, or apoptosis-sensitizing pathways. This is more complex than binary immunohistochemistry, but it may also create opportunities for better combination treatment. CDH3-high tumors may be particularly susceptible to strategies paired with receptor tyrosine kinase inhibition, immune redirection, metabolic disruption, or apoptosis induction.

Several future directions are especially important. Single-cell and spatial multi-omics should define which cellular states truly depend on CDH3 and whether those states are stable or treatment-responsive. Dynamic assessment of membrane localization and adhesion-complex composition may clarify when CDH3 abundance is biologically actionable. Multimodal liquid biopsy integrating soluble cadherin-3, extracellular-vesicle RNA, and circulating protein signatures could improve monitoring and patient selection. Clinical development should also move beyond “CDH3 positive versus negative” toward mechanism-informed trial design, in which ADCs, bispecifics, radioimmunotherapy, or combination regimens are matched to the relevant CDH3 biology in each tumor setting.

## Conclusions and perspectives

CDH3 is now recognized as a translationally relevant node in cancer biology. The accumulated evidence shows that P-cadherin can shape tumor behavior through multilayer regulation, signaling amplification, collective migration, stem-like and metabolic adaptation, and microenvironmental coupling. In many epithelial tumors, particularly breast, lung, gastrointestinal, pancreaticobiliary, ovarian, and brain tumors, CDH3 is linked to aggressive disease and poor outcome. In melanoma, bladder carcinoma, hepatocellular carcinoma, and other selected contexts, however, CDH3 can also restrain invasion or acquire different meanings depending on localization and cadherin background.

This context dependence is not a weakness of the field; it is the key issue that future translational work must resolve. For pathology, it means that abundance alone is unlikely to be sufficient and that membrane localization, spatial heterogeneity, and coexisting cadherin states should be incorporated whenever feasible. For therapeutics, it means that modality selection will need to follow biology: some tumors may be best suited to biomarker use, others to drug delivery, and others to combination strategies that exploit CDH3-associated signaling or multicellular organization.

The most productive way forward is not to ask whether CDH3 is universally oncogenic, but to identify the conditions under which it becomes biologically and therapeutically actionable. The field now has a strong enough foundation to pursue CDH3-guided diagnostics and targeted therapies, but success will depend on better spatial and functional biomarker frameworks, more representative model systems, and biomarker-driven clinical development. If those gaps can be addressed, CDH3 may become a clinically useful bridge between cancer cell-state biology and precision translational oncology.
